# Fbxw7 Controls Angiogenesis by Regulating Endothelial Notch Activity

**DOI:** 10.1371/journal.pone.0041116

**Published:** 2012-07-27

**Authors:** Nanae Izumi, Christian Helker, Manuel Ehling, Axel Behrens, Wiebke Herzog, Ralf H. Adams

**Affiliations:** 1 Max-Planck-Institute for Molecular Biomedicine, Department of Tissue Morphogenesis, and University of Münster, Faculty of Medicine, Muenster, Germany; 2 University of Muenster, Faculty of Biology, and Max-Planck-Institute for Molecular Biomedicine, Angiogenesis Laboratory, Muenster, Germany; 3 Mammalian Genetics Laboratory, CRUK London Research Institute, Lincoln’s Inn Fields Laboratories, London, United Kingdom; Medical College of Wisconsin, United States of America

## Abstract

Notch signaling controls fundamental aspects of angiogenic blood vessel growth including the selection of sprouting tip cells, endothelial proliferation and arterial differentiation. The E3 ubiquitin ligase Fbxw7 is part of the SCF protein complex responsible for the polyubiquitination and thereby proteasomal degradation of substrates such as Notch, c-Myc and c-Jun. Here, we show that Fbxw7 is a critical regulator of angiogenesis in the mouse retina and the zebrafish embryonic trunk, which we attribute to its role in the degradation of active Notch. Growth of retinal blood vessel was impaired and the Notch ligand Dll4, which is also a Notch target, upregulated in inducible and endothelial cell-specific *Fbxw7* ^iECKO^ mutant mice. The stability of the cleaved and active Notch intracellular domain was increased after siRNA knockdown of the E3 ligase in cultured human endothelial cells. Injection of *fbxw7* morpholinos interfered with the sprouting of zebrafish intersegmental vessels (ISVs). Arguing strongly that Notch and not other Fbxw7 substrates are primarily responsible for these phenotypes, the genetic inactivation of Notch pathway components reversed the impaired ISV growth in the zebrafish embryo as well as sprouting and proliferation in the mouse retina. Our findings establish that Fbxw7 is a potent positive regulator of angiogenesis that limits the activity of Notch in the endothelium of the growing vasculature.

## Introduction

The Notch pathway is a versatile signaling system that controls functions as diverse as cell proliferation, differentiation and tissue patterning in a wide range of organs and animal species [1]–[3]. In the developing vasculature, Notch promotes arterial differentiation and inappropriate activation of this pathway can lead to the formation of arteriovenous (AV) shunts [4]. Notch is also an important negative regulator of endothelial sprouting and proliferation. Tip cells, the filopodia-extending endothelial cells (ECs) at the distal (leading) end of growing sprouts, express elevated levels of the Notch ligand Delta-like 4 (Dll4). This, in turn, leads to the activation of Notch receptors in adjacent stalk cells, which form the base of the sprout, and suppresses tip cell behavior in these ECs [5]–[7]. In line with this model, an excessive number of tip cells is formed in haploinsufficient (heterozygous) *Dll4* mutants or in response to the inhibition of Dll4-Notch interactions [8]–[10]. Jagged1, a second transmembrane Notch ligand that is highly expressed in stalk ECs, can interfere with Dll4-Notch signaling and therefore acts as a positive regulator of endothelial sprouting in the developing dermis and retina [11]. The role of Jagged1, Dll4 and Notch is not exclusively confined to tip and stalk cells, but also controls endothelial proliferation throughout the angiogenic vasculature [8], [11], [12].

The examples above show that the levels of Notch activation in the growing vasculature need to be carefully controlled in space and time. Among other mechanisms, the degradation of activated receptors can limit the duration of signaling and thereby control the spatiotemporal activity of pathways [13], [14]. In the case of Notch receptors, ligand binding triggers the proteolytic release of the Notch intracellular domain (NICD), which is then translocated to the nucleus and binds a transcriptional complex containing the transcription factor RBP-Jκ [1], [2]. NICD lifetime is controlled by polyubiquitination and thereby proteasomal degradation, which involves Fbxw7, an F-box protein and the substrate-recognizing component of an SCF (SKP1, CUL1, F-protein)-type E3 ubiquitin ligase [15], [16]. Indicating important roles of Fbxw7 in vascular development, global knockout mice were previously found to be embryonic lethal, strongly growth retarded at E10.5, and showed malformed vessels in the head and yolk sac [17], [18]. Upregulated expression of several Notch target genes in a variety of different embryonic tissues argued that the mutant phenotype was indeed linked to an increased activation of the Notch pathway. However, the question whether Fbxw7 regulates angiogenesis indeed through Notch or other substrates was not investigated so far. Moreover, as the Notch pathway functions in many embryonic tissues such as the heart, neural tube and somites [17], [18], it has remained unclear whether Fbxw7 activity is required in the endothelium or, possibly, in other cell types.

Here, we report the generation of EC-specific and inducible *Fbxw7* mutant mice, which allowed us to investigate the function of the E3 ligase in the growing retinal vasculature, a model system that has been highly useful for the characterization of Notch function [8], [12]. In a second approach, we interfered with zebrafish *fbxw7* expression to characterize the role of the gene during development of the intersegmental vessels in the embryonic trunk, another commonly used experimental model of angiogenesis [19], [20]. Our findings establish that Fbxw7 is indeed an important regulator of endothelial cell behavior in angiogenesis. Impairing Notch activity in both loss-of-function settings mentioned above was sufficient to restore angiogenic sprouting, which indicates that these phenotypes were predominantly caused by excessive Notch activation and not by other Fbxw7 substrates.

## Results

### Inducible Targeting Fbxw7 of in the Vascular Endothelium

**Figure 1:**
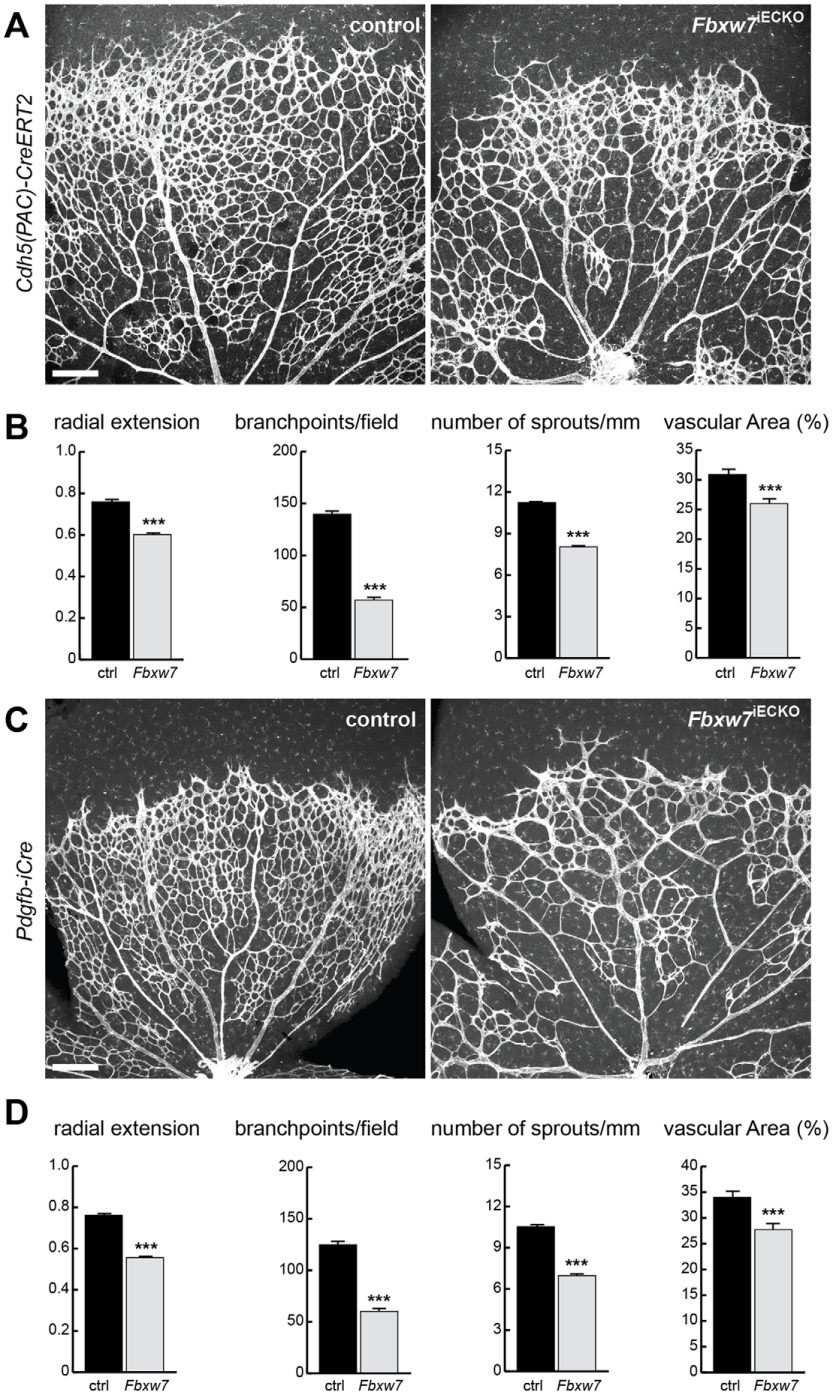
Inducible targeting of the *Fbxw7* gene in retinal ECs. Confocal images showing the organization of the *Fbxw7* ^iECKO^ mutant and littermate control retinal vasculature at postnatal day 6 (P6) after whole-mount Isolectin B4 staining (A and C). Postnatal, EC-specific loss-of-function mutants were either generated with the *Cdh5(PAC)-CreERT2* (A, B) or *Pdgfb-iCre* (C, D) transgenics. Radial extension, branchpoint number, sprouts, and vascular area (B and D) were quantitated (control, n = 7; *Fbxw7* ^iECKO^ n = 6). Error bars indicate SEM. P value (***) <0.00001. Scale bar is 200 µm for all microscopic images.

For functional studies in the postnatal endothelium, mice carrying a loxP-flanked version of the *Fbxw7* gene [21] were combined with *Cdh5(PAC)-CreERT2* [22], [23] or *Pdgfb-iCre* [24] transgenics. Following the postnatal administration of tamoxifen (see Material and Methods), the resulting *Fbxw7* ^iECKO^ mutants generated with either one of these inducible Cre lines displayed very similar phenotypes in the retinal vasculature (Figure 1A, 1C; Figure S1). Angiogenic growth was strongly impaired, which led to a smaller number of vessel branch points, fewer sprouts, a reduction in the area covered by ECs, and delayed extension of the retinal vasculature towards the periphery (Figure 1B, 1D). Both genetic mouse models consistently showed that Fbxw7 is an important positive regulator of angiogenic growth. Moreover, the EC-specific inactivation of *Fbxw7* with tamoxifen-inducible *Cdh5(PAC)-CreERT2* and *Pdgfb-iCre* transgenics demonstrated that the activity of the gene product is indeed cell-autonomously required in endothelial cells.

**Figure 2:**
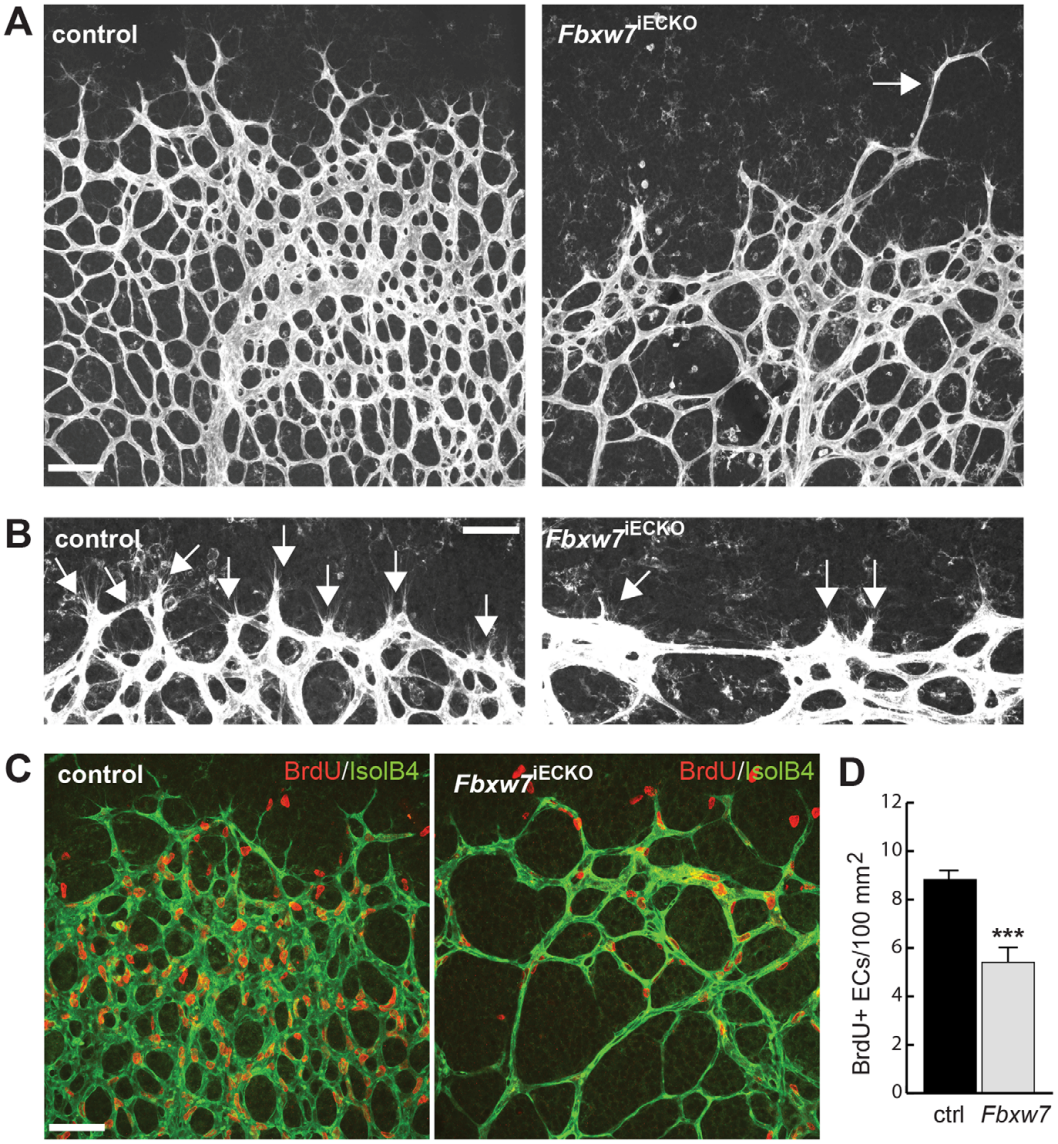
Fbxw7 promotes endothelial sprouting and proliferation. Morphology of the growing (peripheral), Isolectin B4-stained retinal vasculature (A) and higher magnification of endothelial sprouts (B) in *Fbxw7* ^iECKO^ mutants and littermate controls, as indicated. While sprouts were reduced in number and predominantly short in the absence of Fbxw7, isolated long protrusions were observed (arrow in A). BrdU labeling (red) showed reduced endothelial proliferation in the Isolectin B4-stained (green, IsolB4) *Fbxw7* ^iECKO^ retinal ECs (C), which is quantitated in (D). Error bars indicate SEM (based on 8 control and 6 *Fbxw7* ^iECKO^ retinas). P value (***) is <0.00001. Scale bars are 100 µm (A, C) and 50 µm (B).

Previous studies on the role of Notch in the vasculature have led to a model in which Dll4 presented by endothelial tips activates Notch in adjacent (stalk) ECs, which suppresses tip cell behavior in this cell population [5]–[7]. Notch signaling has been also implicated in the dynamic switching of ECs that compete for the tip cell position in growing sprouts [25]. While *Fbxw7* ^iECKO^ mutant endothelial sprouts at the distal edge of the growing retinal vascular plexus were generally short or absent (Figure 2A, 2B), a small fraction of mutant sprouts was longer than normal and extended beyond the edge of the vascular plexus without connecting to other vessels (Figure 1A, 1C, 2A). This phenotype might indicate that the competition of ECs for the tip position or the incorporation of tip cells in new vascular connections were impaired in *Fbxw7* ^iECKO^ mutants. Endothelial proliferation is another important aspect of angiogenic growth that is strongly controlled by the Notch pathway [8], [12]. BrdU labeling showed that the number of proliferating ECs was markedly reduced in *Fbxw7* ^iECKO^ mutants compared to littermate controls (Figure 2C, 2D). Likewise the siRNA mediated knockdown of *Fbxw7* expression in human umbilical vein endothelial cells (HUVECs) led to a significant reduction in cell proliferation (Figure S2). Previous work has also identified the Notch pathway as a positive regulator of arterial differentiation [26], [27]. Indeed, the recruitment of α-smooth muscle actin (αSMA)-positive cells, a feature that is most prominent in the arterial branch of the developing vasculature, was enhanced in *Fbxw7* ^iECKO^ mutants (Figure S2). Key aspects of the *Fbxw7* ^iECKO^ phenotype described here were reproduced by NICD overexpression in the postnatal endothelium (Figure S3). For this purpose, *Pdgfb-iCre* transgenics were bred into a heterozygous or homozygous background of *Gt(ROSA)26Sor* ^tm1(Notch1)Dam/J^ mice [28], which enable Cre-dependent expression of the active Notch1 intracellular domain. Following postnatal administration of tamoxifen, the resulting mutants showed reduced vascular outgrowth, lower vessel density and extension of αSMA-positive vessel beds (Figure S3).

### Enhanced Endothelial Notch Signaling in the Absence of Fbxw7

**Figure 3:**
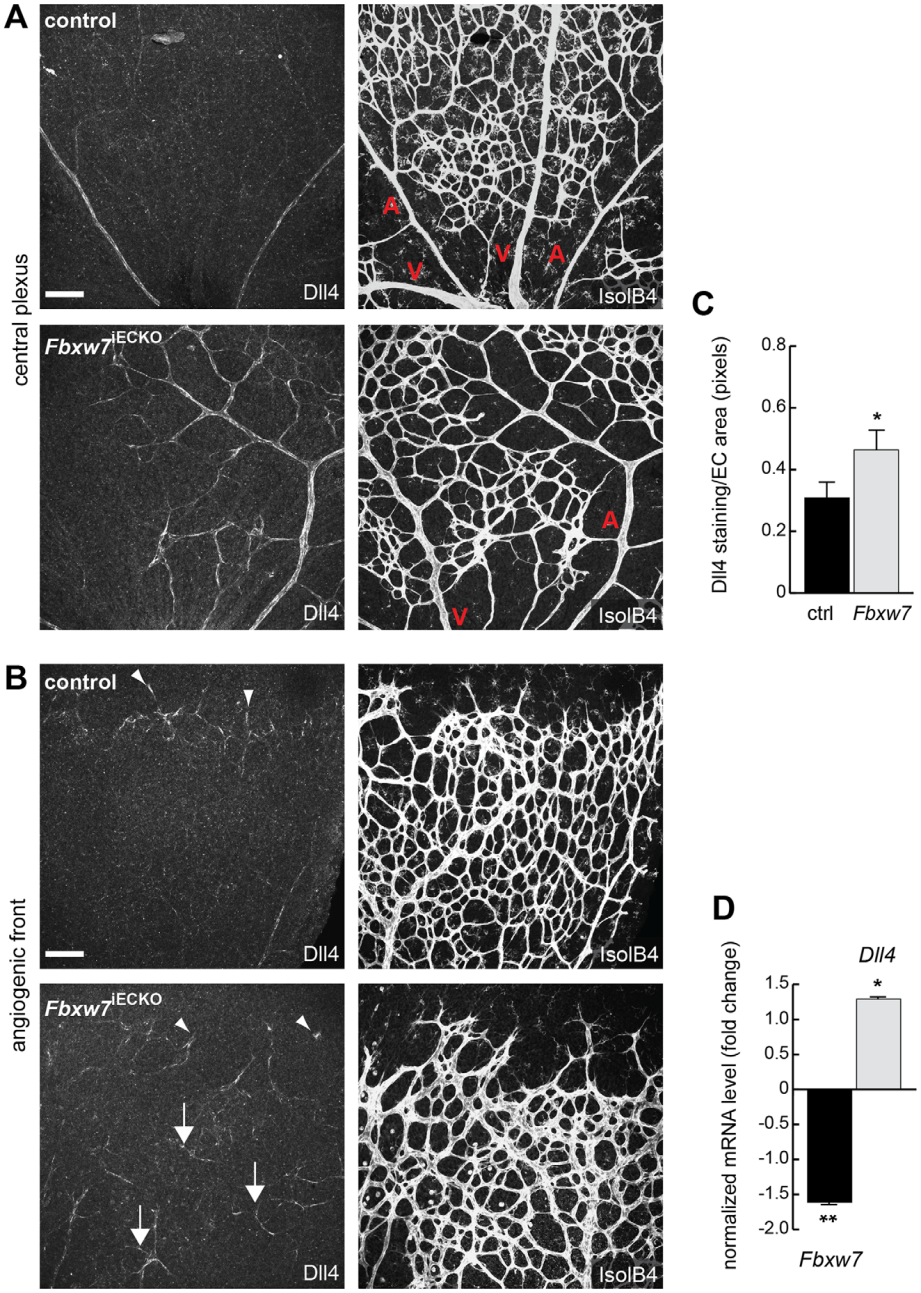
Targeting of *Fbxw7* induced the upregulation of Dll4 expression. Anti-Dll4 and Isolectin B4 (IsolB4)-stained retinal whole-mounts of *Fbxw7* ^iECKO^ and littermate control retinas, as indicated (A, B). Arrowheads in (B) mark Dll4+ peripheral sprouts at the edge of the growing plexus, arrows indicate upregulated Dll4 in *Fbxw7* ^iECKO^ retinal capillaries. Quantitative analysis (with Volocity 5; n = 3 for each group) of image data confirmed elevated Dll4 levels (number of pixels) in the mutant endothelium (C). Likewise, quantitative RT-PCR analysis showed reduced *Fbxw7* expression but upregulated *Dll4* transcript levels in P6 *Fbxw7* ^iECKO^ lungs (D). Expression of the *Cdh5* gene was used for normalization. Error bars indicate SEM. P values are indicated as ** (<0.001) and * (p<0.05). Scale bars are 100 µm.

Dll4 is not only an important activator of Notch in the vasculature, but the ligand is also a strong transcriptional target downstream of activated Notch receptors [5]–[7]. Thus, Dll4 immunofluorescence can provide useful information about the activation of Notch in the endothelium. While anti-Dll4 immunofluorescence labeled arteries and sprouting endothelial cells at the growing edge of the control retinal vasculature, staining extended from arteries into the peri-arterial capillary plexus in *Fbxw7* ^iECKO^ mice (Figure 3A). Moreover, many Dll4-positive ECs were found in mutant capillaries behind the angiogenic front, an area that showed low Dll4 immunofluorescence in control retinas (Figure 3B, 3C). qPCR on total RNA isolated from lungs, a tissue that was chosen because of its high EC content of about 20% of all cells, confirmed that *Dll4* expression was also elevated at the transcript level in *Fbxw7* ^iECKO^ mutants (Figure 3D).

**Figure 4:**
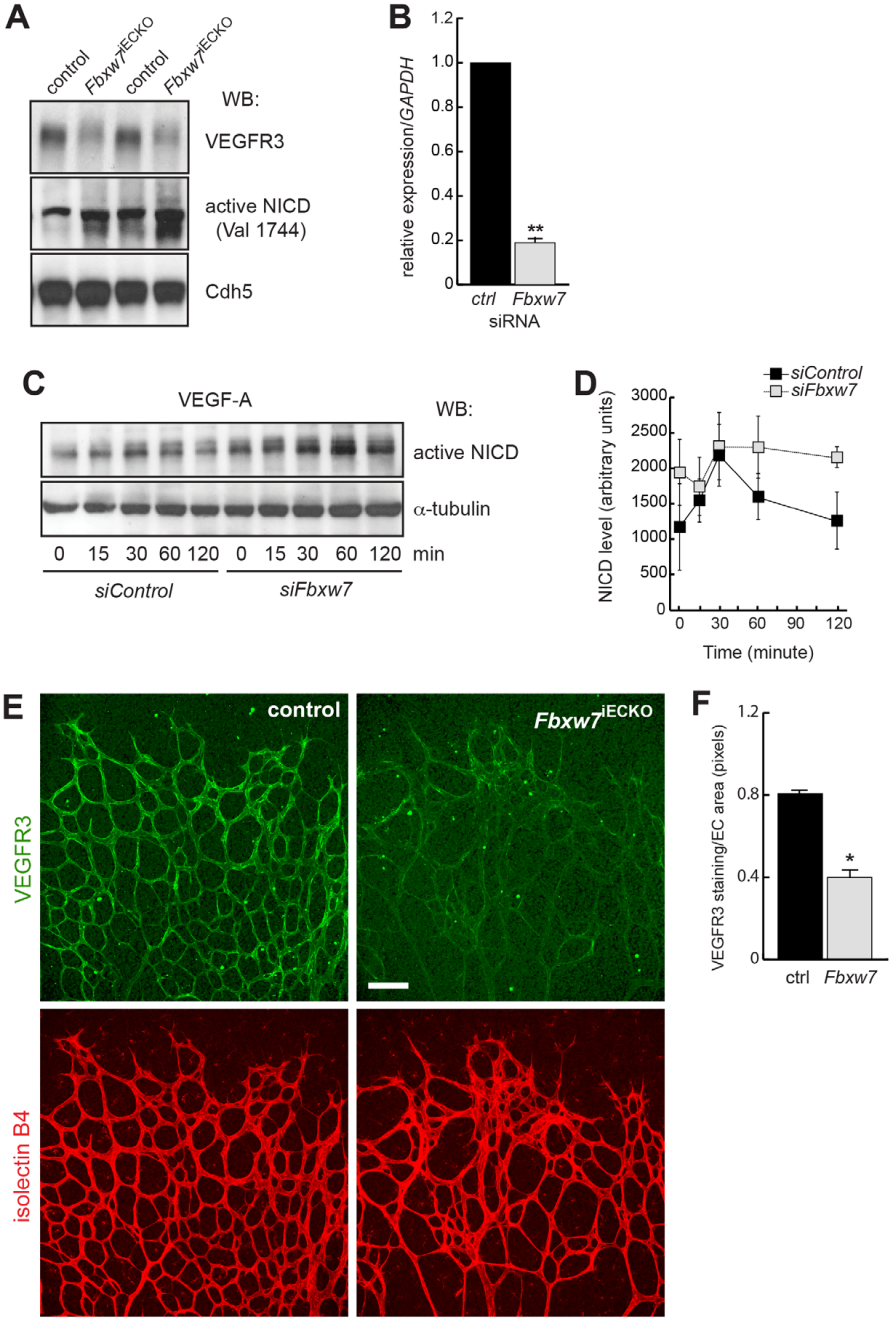
Fbxw7 controls endothelial Notch signaling. Protein extracts prepared from P6 control (lanes 1 & 3 from the left) and *Fbxw7* ^iECKO^ lungs (lanes 2 & 4) showed increased amounts of active Notch1 (NICD-Val1744) and reduced VEGFR3 protein (A). VE-Cadherin (Cdh5) was used for normalization. Relative *Fbxw7* mRNA expression in HUVECs transfected with *siFbxw7* relative to *siControl* siRNAs (n = 3 per group) (B). Human *GAPDH* was used for normalization. VEGF-A stimulation (50 ng/ml for the indicated time periods) led to a transient accumulation of active Notch (NICD) in HUVECs transfected with *siControl*. NICD levels were upregulated after *Fbxw7* silencing (C). Western blot data (band intensity in pixels) was quantified with ImageJ; the graph shows average values from 3 independent experiments (D). Alpha-tubulin was used for normalization. Confocal images showing the pronounced reduction of VEGFR3 protein (green) in the endothelium (Isolectin B4; red) of the P6 *Fbxw7* ^iECKO^ retina (E). Scale bar is 100 µm. Quantitative analysis (with Volocity 5; n = 3 for each group) of image data confirmed reduced VEGFR3 levels (number of pixels) in mutant ECs (F). Error bars (in D and F) indicate SEM. P values are indicated as ** (<0.001) and * (p<0.05).

To directly investigate whether the levels of NICD were increased in *Fbxw7* ^iECKO^ mice, we performed Western blotting with mutant and control lung lysates (Figure 4A). This assay showed that *Fbxw7* mutant samples contained more cleaved, active NICD than controls. Likewise, following knockdown of *Fbxw7* expression by siRNA in cultured HUVECs, baseline NICD levels in *siFbxw7* cells were substantially higher than in control siRNA-treated cells (*siControl*). Interestingly, addition of VEGF-A to the latter led to a transient increase in NICD at 30 minutes followed by a gradual decrease at 60 and 120 minutes after stimulation (Figure 4C, 4D). In *siFbxw7* HUVECs, NICD also increased in response to VEGF-A, but levels stayed high and remained significantly above what was seen in *siControl* cells. Thus, reduced expression of Fbxw7 in cultured endothelial cells increased the stability of the active Notch intracellular domain.

The role of the Notch pathway in angiogenesis is strongly linked to VEGF signaling. In stalk ECs, active Notch is thought to suppress or dampen VEGF receptor expression, which contributes to the distinct behaviors of leading (tip) and trailing (stalk) cells within vascular sprouts [5]–[7]. In the addition to signaling by VEGF-A and VEGFR2, VEGFR3, the receptor for VEGF-C and VEGF-D, is an important regulator of angiogenesis and its expression is suppressed by active Notch [12]. In line with enhanced Notch signaling in the endothelium of *Fbxw7* ^iECKO^ mice, immunofluorescence showed that VEGFR3 protein expression was strongly reduced (Figure 4E, 4F).

### Fbxw7 Controls Intersegmental Vessel Growth in Zebrafish

**Figure 5:**
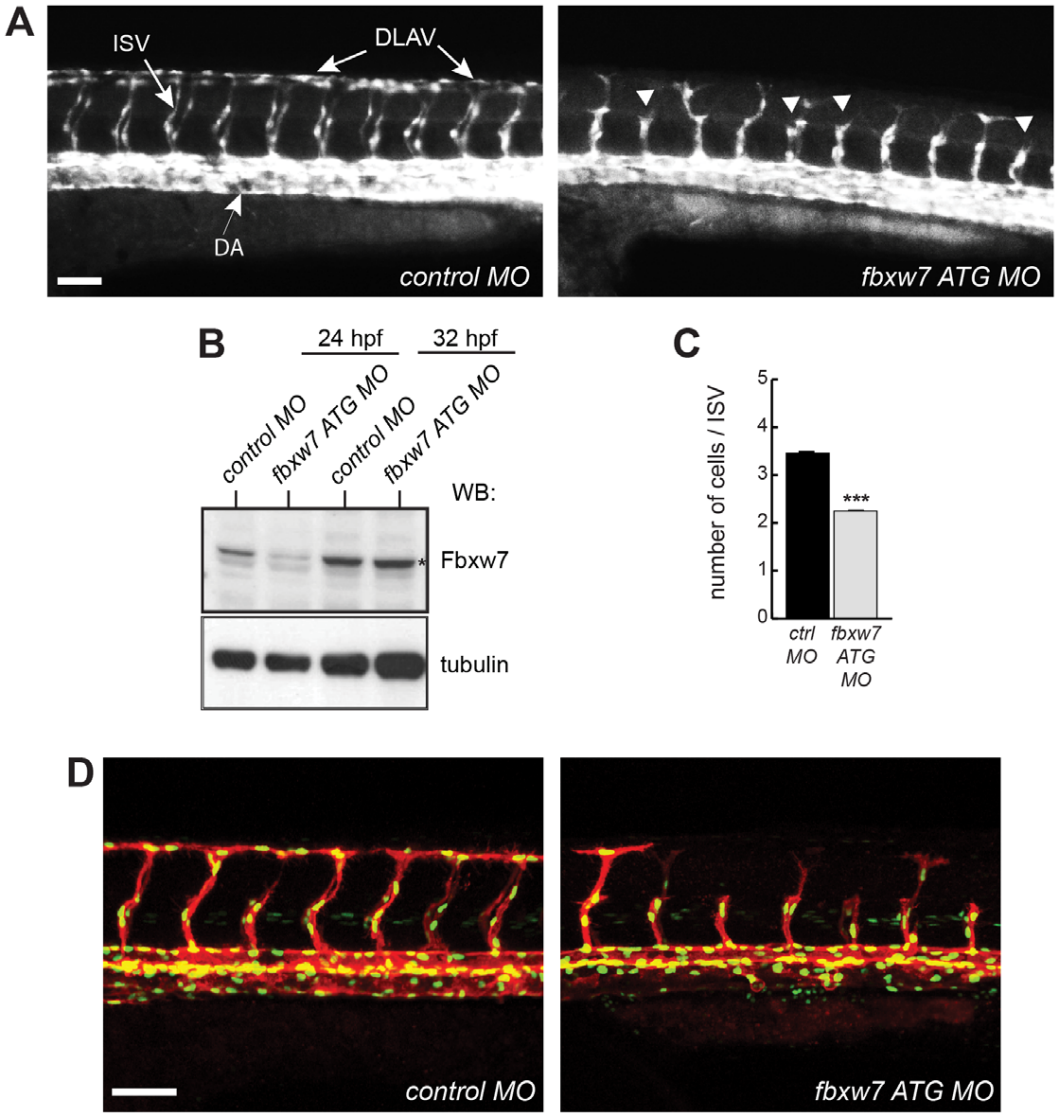
Impaired ISV growth after knockdown of zebrafish *fbxw7* expression. Following injection with control morpholino (*control MO*) or fbxw7 transcription blocking morpholino (*fbxw7 ATG MO*), the fluorescent vasculature of *Tg(kdrl:EGFP)^s843^* transgenic embryos was examined by live microscopy. Outgrowth of ISVs from the dorsal aorta (DA) and formation of the DLAV (as indicated in the left image) were impaired in *fbxw7* morphant embryos (arrowheads) at 32 hpf (A). Scale bar is 70 µm. Protein extracts from control and *fbxw7 ATG MO* embryos at 24 hpf and 32 hpf were analyzed by Western blotting (B). Note transient reduction of the Fbxw7 band (asterisk). Tubulin was used as loading control. Quantification of the number of ECs per ISV in control MO and *fbxw7 ATG MO* injected embryos at 32 hpf (based on 3 independent experiments) (C). Proliferation of endothelial cells at 32 hpf in ISVs of *Tg(fli1a:nEGFP)^y7^* x *Tg(kdrl:HsHRAS-mCherry)^s896^* double transgenic embryos injected with *control MO* or *Fbxw7 ATG MO* (D). Error bars indicate SEM. P value (*) in (C) is <0.05.

The VEGF and Notch pathways are also important regulators of angiogenesis in the zebrafish embryo, which has been shown for the growth of intersegmental vessels from the dorsal aorta in the embryonic trunk [29], [30]. To investigate whether zebrafish *fbxw7* might control this process, we reduced the expression of this gene with antisense morpholino oligonucleotides (MOs) that interfere with transcript splicing or translation, respectively (see Materials and Methods; Figure S4). The vasculature in these experiments was visualized by endothelial expression of green fluorescent protein in a *Tg(kdrl:EGFP)^s843^* transgenic background [31]. Microinjection of control or either of the two *fbxw7* MOs at the chosen concentration did not interfere with normal growth and general development of the embryos (Figure S4). Significant downregulation of Fbxw7 protein was seen at 24 hours post fertilization (hpf), which was, however, transient and did not persist at 32 hpf (Figure 5B). Despite the temporary nature of the *fbxw7* knockdown, ISV growth was defective in morphant embryos analyzed at 32 hpf (Figure 5A, 5B; Figure S4). Intersegmental vessels, which were fully extended and had started to form the dorsal longitudinal anastomotic vessel (DLAV) in control MO-injected embryos, had only grown halfway up to the myoseptum and the DLAV was absent in *fbxw7* morphants (Figure 5A, Movies S1 and S2). To analyze the proliferation of endothelial cells during ISV outgrowth, control or *fbxw7* MOs were injected into double transgenic embryos expressing a nuclear green fluorescent protein reporter *Tg*(*flia:nEGFP*)^y7^ together with a membrane-anchored red fluorescent protein, encoded by the *Tg*(*kdrl:HsHRAS-mCherry*)^s896^ transgene, in the endothelium [32], [33] (Figure 5D). Counting of endothelial nuclei in growing ISVs in these embryos showed that the knockdown of *fbxw7* expression led to pronounced reduction in the number of intersegmental ECs (Figure 5C, 5D), which is reminiscent of the phenotype seen in zebrafish embryos with enhanced Notch signaling [34].

### The Role of Fbxw7 in Angiogenesis is Mainly Mediated by Notch

**Figure 6:**
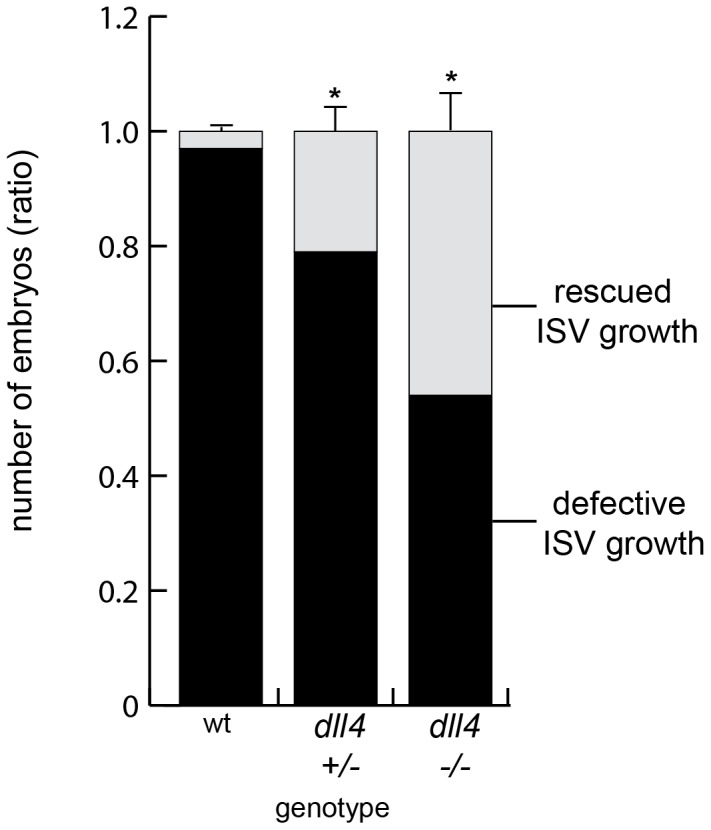
Impaired Notch signaling alleviates the *fbxw7* morphant phenotype in zebrafish. Offspring resulting from an intercross of heterozyogous *dll4^j16e1^* mutants were injected with *fbxw7 ATG MO* and the ISV morphology was analyzed at 32 hpf. The fraction of embryos showing normal ISV growth (light grey) was highest in a *dll4* homozygous background. In contrast, *fbxw7* silencing led to defective ISV growth (black) in almost all *dll4* wild-type embryos. The ratio of ISV phenotypes within each genotype group was calculated (based on 3 independent experiments). Error bars reflect SEM. P value (*) is <0.05.

Fbxw7 is responsible for the polyubiquitination of multiple substrates apart from Notch [15], [16]. To address whether Notch or other target proteins of Fbxw7 were responsible for the angiogenesis defects seen in zebrafish ISVs, we injected *fbxw7* or control morpholinos into zebrafish mutant embryos with a defective *dll4* gene. These *dll4* mutants display enhanced EC sprouting and proliferation [30] and thereby resemble the vascular phenotypes caused by the full or partial inhibition of Dll4 function in mice [8]–[10]. We found that the impaired angiogenesis seen in the ISVs of *fbxw7* morphants was partially rescued in the *dll4* homozygous mutant background (Figure 6). Whereas defective ISVs were seen in 97% of morphant embryos with a wild-type *dll4* gene, intersegmental vessel growth was restored in 21% of heterozygous and 46% of homozygous *dll4* mutants.

**Figure 7:**
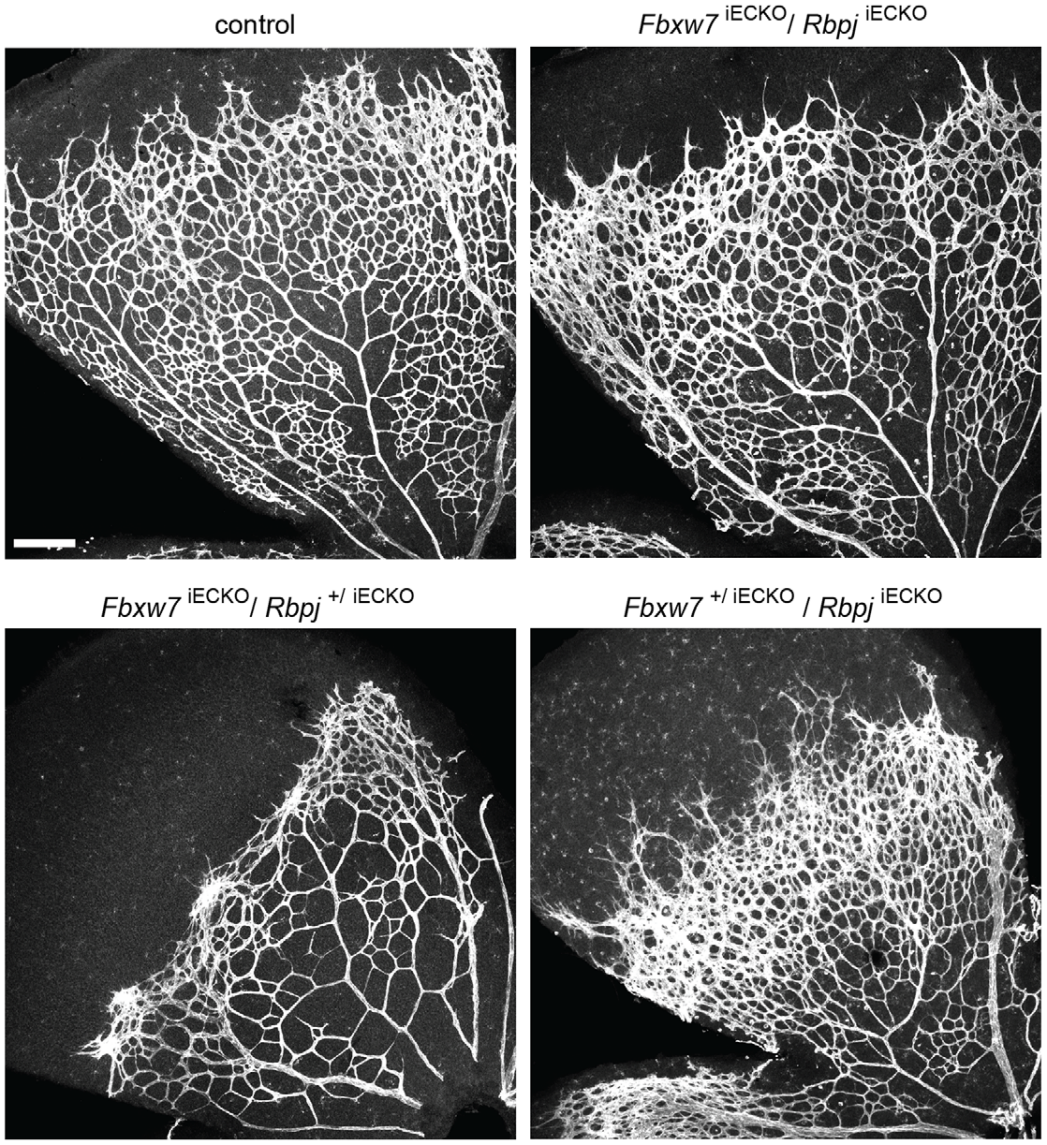
*Rbpj* inactivation reverses the impaired vascular growth of *Fbxw7* mutant mice. Whole-mount Isolectin B4 staining of the retinal vasculature in P6 control, *Fbxw7* ^iECKO^/*Rbpj* ^+/iECKO^ (*Fbxw7* homozygous & *Rbpj* heterozygous EC-specific KO), *Fbxw7* ^iECKO^/*Rbpj* ^iECKO^ (*Fbxw7* & *Rbpj* EC-specific double KO), and *Fbxw7* ^+/iECKO^/*Rbpj* ^iECKO^ (*Fbxw7* heterozygous & *Rbpj* homozygous EC-specific KO), mice, as indicated. Inactivation of both *Rbpj* alleles enhanced EC proliferation and sprouting, and restored retinal angiogenesis in the *Fbxw7* mutant background. Scale bar represents 200 µm. Three independent experiments were performed with similar results.

A similar rescue experiment was conducted in the mouse by combining *Cdh5(PAC)-CreERT2* transgenic mice [23] with conditional, loxP-flanked alleles of *Fbxw7* [21] and *Rbpj* [35] (the gene encoding RBP-Jκ) (Figure 7). Tamoxifen-induced targeting *Fbxw7* in the postnatal endothelium in a wild-type (data not shown) or *Rbpj* heterozygous background strongly reduced retinal angiogenesis (Figure 7). Inactivation of the *Rbpj* gene in the retinal endothelium led to enhanced endothelial sprouting and proliferation reminiscent of other Notch pathway mutants. Remarkably, the combined inactivation of both *Fbxw7* and *Rbpj* restored angiogenic growth in the resulting double mutants indicating that the *Fbxw7* ^iECKO^ phenotype is indeed primarily a consequence of excessive Notch signaling (Figure 7). Likewise, short-term pharmacological inhibition of Notch signaling with the γ-secretase inhibitor DAPT (see Material and Methods), which blocks Notch cleavage and activation, also reversed the *Fbxw7* retinal phenotype (Figure S5). These rescue experiments demonstrate that the biological role of Fbxw7 as a positive regulator of angiogenesis is critically mediated by the degradation of Notch.

## Discussion

Previous work has shown that Fbxw7, a component of an SCF-type ubiquitin ligase complex, controls several aspects of cardiovascular development in the mouse. Targeted inactivation of the gene led to embryonic lethality at midgestation and severe growth retardation, which has been attributed to defects in the mutant hematopoietic system as well as impaired cardiovascular development [17], [18]. Due to the interdependent nature of morphogenetic processes in the embryonic placenta, heart and vasculature [36], [37], these findings left substantial uncertainty with regard to the exact role of Fbxw7 in the regulation of angiogenic blood vessel growth. Here we have targeted the *Fbxw7* gene in the endothelium of the postnatal mouse with an inducible and cell type-specific approach. Our analysis of the postnatal retinal vasculature, a well-established model system of angiogenesis, demonstrated that the activity of Fbxw7 is indeed critically required in endothelial cells. EC-specific *Fbxw7* ^iECKO^ mutants displayed a pronounced reduction in endothelial sprouting and proliferation indicating that the protein is an important positive regulator of both processes.

Previous work has identified active Notch as a substrate of the Fbxw7-containing ubiquitin ligase complex [15]. Notch target genes were also found to be upregulated in embryos with a full *Fbxw7* gene knockout [17], [18] and, accordingly, it has been proposed that the vascular phenotype of these mutants is caused by defective Notch degradation. However, in addition to active Notch, numerous other Fbxw7 substrates have been identified. Examples are the cell cycle regulator cyclin E, the proto-oncogenes c-Myc and c-Jun, or the sterol regulatory element binding protein (SREBP) [15], [16], all of which might be relevant in the context of the vascular defects seen in the Fbxw7-deficient embryos. To address this important mechanistic question directly, we tested whether interfering with Notch signaling can circumvent the angiogenic growth block seen in *Fbxw7* loss-of-function models. Indeed, inactivation of the *dll4* gene was sufficient to restore angiogenesis in the trunk of a large fraction of *fbxw7*-silenced zebrafish embryos. In the mouse retina, inactivation of the *Rbpj* gene or pharmacological inhibition of Notch signaling with DAPT were sufficient to reestablish substantial levels of angiogenic growth in the *Fbxw7* ^iECKO^ mutant background. Thus, our new data directly demonstrate that excessive Notch activation is a predominant cause of compromised sprouting and proliferation in *Fbxw7* loss-of-function models. This finding does not rule out that other Fbxw7 substrates might play minor or functionally distinct roles in the regulation of endothelial cell behavior or other aspects of vascular morphogenesis.

The Notch pathway is a critical regulator of angiogenesis and has been linked to VEGF signaling. Expression of the ligand Dll4 is induced by VEGF-A and, conversely, Dll4-mediated activation of Notch is thought to downregulate the expression of VEGF receptors in ECs, which generates a negative feedback loop that dampens VEGF-triggered angiogenesis [5]–[7]. A second Notch ligand, Jagged1, opposes Dll4 and thereby promotes angiogenic growth in the skin and retina [11]. As the behavior of sprouting endothelial cells is highly dynamic, which involves positional shuffling and competition for the tip position during sprouting [25], the spatiotemporal pattern of Notch activation needs to be equally flexible. In addition to the regulation of Notch receptor and ligand expression, modulating the lifetime of the proteolytically cleaved (active) NICD provides another level of control [38], [39]. The importance of this regulatory mechanism is highlighted by our finding that Fbxw7-deficiency in ECs led to increased NICD stability and compromised endothelial sprouting *in vivo*. While we have not investigated the active positional switching capability of individual *Fbxw7* ^iECKO^ endothelial cells, our phenotypic data indicates that prolonging the lifetime of endogenously generated NICD leads to a general slowdown in angiogenic growth. This might reflect that most or all ECs - irrespective of whether they are positioned in the tip, stalk or in capillaries - are at least transiently exposed to a certain level of Notch activation, which is enhanced or prolonged in *Fbxw7* mutants.

The overexpression of active Notch can cause vascular malformations such as the development of AV shunts [4]. As our findings show that Fbxw7 deficiency can lead to the accumulation of NICD in endothelial cells, it appears worthwhile to investigate whether mutations in the *Fbxw7* gene are linked to human cardiovascular diseases. Likewise, it will be important to gain a better understanding of the modifications that mark active Notch receptors for polyubiquitination. Here, the regulated phosphorylation of Fbxw7 recognition sites (degrons) or other posttranslational modifications of the NICD are likely to be of central importance [38], [39]. It also remains to be addressed whether the controlled modulation of Fbxw7 expression or activity can be utilized for therapeutic purposes in eye diseases and other settings where it is desirable to suppress angiogenic blood vessel growth.

## Materials and Methods

### Inducible Mutant Mice

Mice carrying a loxP-flanked (floxed) *Fbxw7* gene [21] were combined with *Cdh5(PAC)-CreERT2* [22], [23] or *Pdgfb-iCre* [24] transgenic lines, respectively. All *Fbxw7* ^iECKO^ mutants shown were obtained with the *Cdh5(PAC)-CreERT2* strain unless indicated otherwise. Tamoxifen-injected Cre-negative littermates were used as controls. For the generation of double mutants, floxed alleles of the *Rbpj* gene [35], floxed *Fbxw7* and the *Cdh5(PAC)-CreERT2* transgene were combined by crossbreeding. To overexpress NICD in the postnatal endothelium, *Pdgfb-iCre* heterozygotes were bred into a heterozygous or homozygous background of *Gt(ROSA)26Sor* ^tm1(Notch1)Dam/J^ transgenics [28]. Detailed protocols for the administration of tamoxifen were published previously [11], [22]. Mutant phenotypes were analyzed at postnatal day 6 (P6) unless other information is provided.

DAPT treatment was performed according to Benedito et al. [11] with minor modifications. Following the tamoxifen injection, pups were twice intraperitoneally injected (at P4 and P5) with 0.1 mg/g γ-secretase inhibitor IX (DAPT; Calbiochem, 565770) and retinas were collected 48 hrs later at P6.

All animal experiments were performed in strict accordance with the relevant laws and institutional guidelines at Cancer Research UK and the Max Planck Institute for Molecular Biomedicine. All protocols were approved by animal ethics committees of Cancer Research UK (UK) and the state of North Rhine-Westfalia (Germany), respectively, and all efforts were made to minimize suffering.

### Whole-mount Immunohistochemistry of the Retinal Vasculature

P6 mouse eyes were fixed in 4% PFA for 2 hrs at room temperature (RT) or on ice. Retinas were blocked in blocking buffer (1% BSA, 0.3% TritonX-100 in PBS) followed by incubation with primary antibodies in Pblec (1 mM CaCl2, 1 mM MgCl2, 0.1 mM MnCl2, 1% TritonX-100 in PBS) for RT-fixed retinas or in 0.5 Pblec (Pblec +0.5% TritonX-100) for samples fixed on ice. Endothelial cells were visualized with biotinylated Griffonia Simplicifolia lectin I (Isolectin B4) (Vector Labs, B1205). The following primary antibodies were used: goat anti-mouse Dll4 (R&D Systems, AF1389), rat anti-mouse VEGFR3/Flt4 (eBioscience, 14-5988), and mouse Cy3-conjugated anti-alpha-smooth muscle actin (Sigma-Aldrich, C6198). The corresponding secondary antibodies were coupled with AlexaFluor 488, 546, or 647 (Invitrogen).

### BrdU Staining of Proliferating Cells

For the detection of proliferating cells, 300 µg of 5-bromo-2′-deoxyuridine (BrdU) (Invitrogen, B23151) was injected intraperitoneally 3 hrs before the animals were humanely killed. Eyes were fixed in 4% PFA for 2 hrs at RT. After Isolectin B4 staining, retinas were re-fixed in 4% PFA for 30 min at room temperature, washed 3 times with PBS, incubated in 50% Formamide/1× SSC (0.15 M NaCl and 0.015 M Na-citrate) [PH 7.0] for 1 hr at 65°C, incubated in 2 N HCl for 30 min in a 37°C water bath, neutralized with 0.1 M Tris-HCl [pH 8.0], blocked in blocking buffer for 2 hrs at room temperature, and incubated overnight with a mouse anti-BrdU antibody (BD Biosciences, 347580). Secondary detection was performed with an Alexa Fluor 546-coupled secondary antibody (Invitrogen, A11003).

### Quantitative Analysis and Imaging

Radial extension, length of vascular growth from the optic nerve, branchpoints, number of sprouts, and vascular area of P6 retinas were quantified as described previously [11]. Student’s t-test was used for statistical analysis.

Whole-mount images of the retinal vasculature and zebrafish embryos were taken with a Leica TCS SP5 confocal microscope using the following objectives: 10×/0.30 NA (Figures 1 and 7), 20×/0.70 NA (Figures 2, 3, and 4), and 20×/0.50 NA (Figure 5). Low magnification images were acquired with a Leica MZ16F stereomicroscope. Images were converted to greyscale in Volocity 5 (Improvision) and managed in Photoshop CS3 (Adobe Systems Inc.).

### Endothelial Cell Culture and siRNA Knockdown Experiments

Human umbilical vein endothelial cells (HUVECs) were purchased from Invitrogen and were maintained in Medium200 (M200) supplemented with low serum growth supplement (LSGS) (Invitrogen). HUVECs at passage 3 to 6 were used for experiments.

For the VEGF-A stimulation assay, HUVECs were transfected with Stealth RNA interference oligonucleotides (Invitrogen) against either control (300 nM) (Negative Medium GC:12935-300, Invitrogen) or human *FBXW7* (mixture of #1, HSS124318 and #3, HSS124320, Invitrogen; 150 nM each) using Oligofectamine (Invitrogen) according to manufacturer’s instructions. At 72 hrs post-transfection, confluent HUVECs were serum-starved in M200 supplemented with 0.5% fetal bovine serum (FCS; Biochrom AG) for 4 hrs. After stimulation with VEGF-A165 (50 ng/ml) (ReliaTech GmbH, 300076L) in M200 supplemented with 0.5% FCS, cells were incubated and collected with Laemmli sample buffer at the indicated time points.

For the extraction of RNA, the RNeasy Mini kit (Qiagen) was used to purify total RNA from HUVECs at 72 hrs post-transfection. 500 ng RNA/reaction were used to generate cDNA using the iScript cDNA Synthesis Kit (BioRad).

To assess the growth for *siControl* and *siFbxw7* HUVECs, cells were detached, diluted and counted with a Neubauer chamber at 72 hrs post-transfection. Six independent transfections were used for quantitation.

### Processing of Mouse Lung Lysates

Lungs were isolated from P6 mice. Left lobes were used for RNA analysis, whereas right lobes were used for protein analysis in individual pups. The RNeasy Mini kit (Qiagen) was used to purify total RNA. 500 ng RNA/reaction were used to generate cDNA using the iScript cDNA Synthesis Kit (BioRad).

For the extraction of protein, lung samples were lysed in RIPA buffer (50 mM Tris/HCl [pH 8.0], 150 mM NaCl, 1% NP-40, 0.1% SDS, and 0.5% sodium deoxycholate) supplemented with protease and phosphatase inhibitor cocktails (Thermo Scientific). Samples were sonicated 4× for 10 sec followed by incubation for 30 min at 4°C. Lysates were then centrifuged for 30 min at 16100×g at 4°C. Supernatants were collected and combined with Laemmli sample buffer.

### Western Blot Analysis

Western blots of lung or HUVEC samples were probed with the following antibodies: rat anti-mouse VEGFR3/Flt4 (eBioscience, 14-5988), rabbit anti-cleaved Notch1 (NICD-Val1744) (Cell Signaling Technology, 2421), rabbit anti-activated Notch1 (NICD) (Abcam, ab8925), rat anti-mouse CD144/Cdh5 (BD Biosciences, 555289), mouse anti-alpha-tubulin (Sigma-Aldrich, T5168), and rabbit anti-Cdc4/Fbxw7 (Millipore, ab10620).

### Quantitative PCR Analysis

Real-time quantitative PCR reactions were performed in duplicate per reaction using the Taqman Gene Expression Assay system (Applied Biosystems). The following Taqman primers were used: *mCdh5* (Mm00486938_m1), *mFbxw7* (Mm00504445_m1), *mDll4* (Mm00444619_m1), *hGAPDH* (FAM Dye/MGB Probe, 4352934), and *hFbxw7* (Hs01015617_m1).

### Zebrafish Strains

Zebrafish were maintained in a recirculating aquaculture system under standard laboratory conditions at 27°C. Embryos were grown at 28.5°C and staged in hours post fertilization according to Kimmel et al. [40]. The transgenic lines used were *Tg(fli1a:nEGFP)^y7^*, *Tg(kdrl:EGFP)^s843^*, *Tg(fli1a:EGFPp)^y1^*, and *Tg(kdrl:HsHRAS-mCherry)^s896^* [31]–[33]. Morpholino antisense oligonucleotides (morpholinos, MO) were obtained for the following sequences (GeneTools LLC): *fbxw7 ATG MO* (translation blocking), 5′ - CCTTGCCCGAGGACTTGGTCATCTT -3′; *fbxw7 Splice MO* (splicing blocking), 5′-GGAAGACAGCAGTTTTACCTTGGAC- 3′ and *control MO* (standard control morpholino) (Gene Tools LLC). Morpholinos (3.3 ng/embryo) were injected into one to two-cell stage embryos. For the validation of the *fbxw7 ATG MO* in zebrafish by Western blot, 20 injected embryos for each experiment were dechorionated manually and, after removal of the yolk, lysed in 40 µl 2× Laemmli sample buffer containing 3% 2-mercaptoethanol. Western blots were probed with rabbit anti-Cdc4/Fbxw7 antibody (Millipore, ab10620). For the validation of the *fbxw7 Splice MO*, cDNA from injected embryos at 7 somite stage (12,5 hpf) was analyzed by PCR with the following primers: *fbxw7 forward*: 5′-CCCAGAGGTTCGATCTTTCA-3′, *fbxw7 reverse*: 5′-AAGGTGCGAAGCCATTCTT-3′. As loading control, PCR with the following beta-actin primers [41] was performed on the same cDNA samples: *forward* 5′-CGAGCAGGAGATGGGAACC-3′ and *reverse*: 5′-CAACGGAAACGCTCATTGC-3′. DNA products generated by PCR were visualized by gel electrophoresis.

### Quantification and Imaging of ISV Sprouts in Zebrafish

To determine changes in ISV cell number following *fbxw7* knockdown, *control MO* and *fbxw7 ATG MO*-injected embryos were examined live or fixed for 2 hrs with 4% PFA at room temperature and analyzed by confocal microscopy (Leica TCS SP5). Confocal stacks and movies were assembled using Imaris Software (Bitplane). From each embryo, the number of cells in four ISVs, located between the 12–16th somite were counted. Endothelial cell nuclei were visualized by transgenic GFP expression using the *Tg(fli1:nEGFP)^y7^*. Five to six embryos were analyzed per injection sample.

For time-lapse microscopy, embryos were dechorionated manually and mounted in agarose embryo arrays as described previously [42]. To prevent movement of embryos, medium was supplemented with 19.2 mg/l (0.0192%) tricaine (ethyl-3 aminobenzoat methane sulfonate salt). Pigmentation was inhibited by adding phenylthiourea to a final concentration of 0.003%.

### Rescue of the fbxw7 Phenotype in Zebrafish

Embryos generated by intercrossing *dll4^j16e1^* heterozygotes were injected at the one cell stage with *fbxw7 ATG MO* morpholino. The injected embryos were fixed at 32 hpf for 2 hrs with 4% PFA at room temperature, analyzed using a Leica M165FC fluorescence microscope, and genotyped individually by PCR and subsequent restriction analysis. Primers used for genotyping were: *dll4^j16e1^* FWD 5′-AGTTTTAGATTGTGCTGG AACATCTTGTATA-3′ and *dll4^j16e1^* REV 5′-GACATATTTTCAATCAAATCCAGTCATGAT-3′. The PCR products were digested with Bpm1.

## Supporting Information

Figure S1 **Defective retinal angiogenesis in** ***Fbxw7*** **loss-of-function mutants.** Confocal images of whole-mount Isolectin B4-stained retinas from P6 *Fbxw7* ^iECKO^ mutants generated with the *Cdh5(PAC)-CreERT2* (A) or *Pdgfb-iCre* (B) transgenic line, respectively. Controls (left panels) are respective littermates. Note the impaired retinal angiogenesis in the absence of endothelial *Fbxw7*. Scale bar is 500 µm.
(TIF)

Figure S2 ***Fbxw7*** **controls HUVEC proliferation and smooth muscle cell recruitment.** Quantitation of cell numbers in cultured *siFbxw7* (*Fbxw7* silenced) or *siControl* HUVECs, as indicated, at 72 hours after siRNA transfection (A). Error bars indicate SEM. P value (**) is <0.001. Confocal image of P9 whole-mount *Fbxw7* ^iECKO^ (right) and control (left) retinas after staining with anti-smooth muscle actin (SMA) antibody (B). Note increased SMA signal in the mutant vasculature. Scale bar represents 500 µm.
(TIF)

Figure S3 **Endothelial overexpression of NICD phenocopies Fbwx7 defects.** Confocal images of P6 control and endothelial cell-specific NICD gain-of-function retinas (A). *Pdgfb-iCre* transgenics were combined with *Gt(ROSA)26Sor* ^tm1(Notch1)Dam/J^ heterozygous (middle column) or homozygous mice (right column). ECs were visualized by Isolectin B4 (green), smooth muscle cells by αSMA immunofluorescence. Bottom panels show higher magnification of insets in the middle row. Arrows indicate extension of αSMA staining into peri-arterial capillary beds. Radial extension of the vascular plexus towards the periphery (B) was reduced in NICD/+ heterozygotes and NICD/NICD homozygotes.
(TIF)

Figure S4 **Vascular defects caused by the knockdown of zebrafish** ***fbxw7*** **.** Bright-field images (A, C) and endothelial fluorescence (B, D) of *Tg(kdrl:EGFP)^s843^* zebrafish embryos at 32 hpf injected with control (*control MO*), *fbxw7* translation-blocking (*ATG MO*) or *fbxw7* splicing-blocking (*Splice MO*) morpholinos, as indicated. The knockdown of *fbxw7* impaired ISV outgrowth and prevented the formation of the DLAV, while the size and general growth of the morphant embryos were unaffected. Scale bar is 200 µm. PCR analysis (E) showing the reduction of *fbxw7* transcripts in zebrafish embryos injected with *Splice MO* in two independent experiments. Beta-actin PCR products were used as loading control.
(TIF)

Figure S5 **Notch inhibition restores vascular growth in** ***Fbxw7*** **mutants.** Confocal images of whole-mount Isolectin B4-stained retinas. Notch inhibition was achieved by administration of the γ-secretase inhibitor DAPT, which interferes with Notch cleavage and signaling, for 48 hrs prior to the isolation of the retinas at P6. DAPT partially restored enhanced sprouting and proliferation in the *Fbxw7* ^iECKO^ vasculature. In contrast, angiogenesis was not increased in vehicle (DMSO)-injected *Fbxw7* ^iECKO^ mutants. The phenotype of DMSO or DAPT-treated control retinas is shown in the upper row. Scale bar is 200 µm.
(TIF)

Movie S1 **ISV outgrowth** ***control MO*** **-injected embryos.** Time-lapse movie of ISV growth in the trunk of a *control MO*-injected *Tg(kdrl:EGFP)^s843^* transgenic embryo from 19 hpf to 42 hpf.
(MOV)

Movie S2 **ISV outgrowth** ***Fbxw7 ATG MO*** **-injected embryos.** Time-lapse movie of ISV growth in the trunk of a *fbxw7 ATG MO*-injected *Tg(kdrl:EGFP)^s843^* transgenic embryo from 19 hpf to 42 hpf. Note stalling of the ISVs and their failure to extend beyond the myoseptum in dorsal direction.
(MOV)
