# Supplementary figures and images for: Fbxw7 Controls Angiogenesis by Regulating Endothelial Notch Activity
Source: PLoS One. 2012 Jul 27;7(7):e41116. doi: 10.1371/journal.pone.0041116 (PMC3407154; doi:10.1371/journal.pone.0041116)

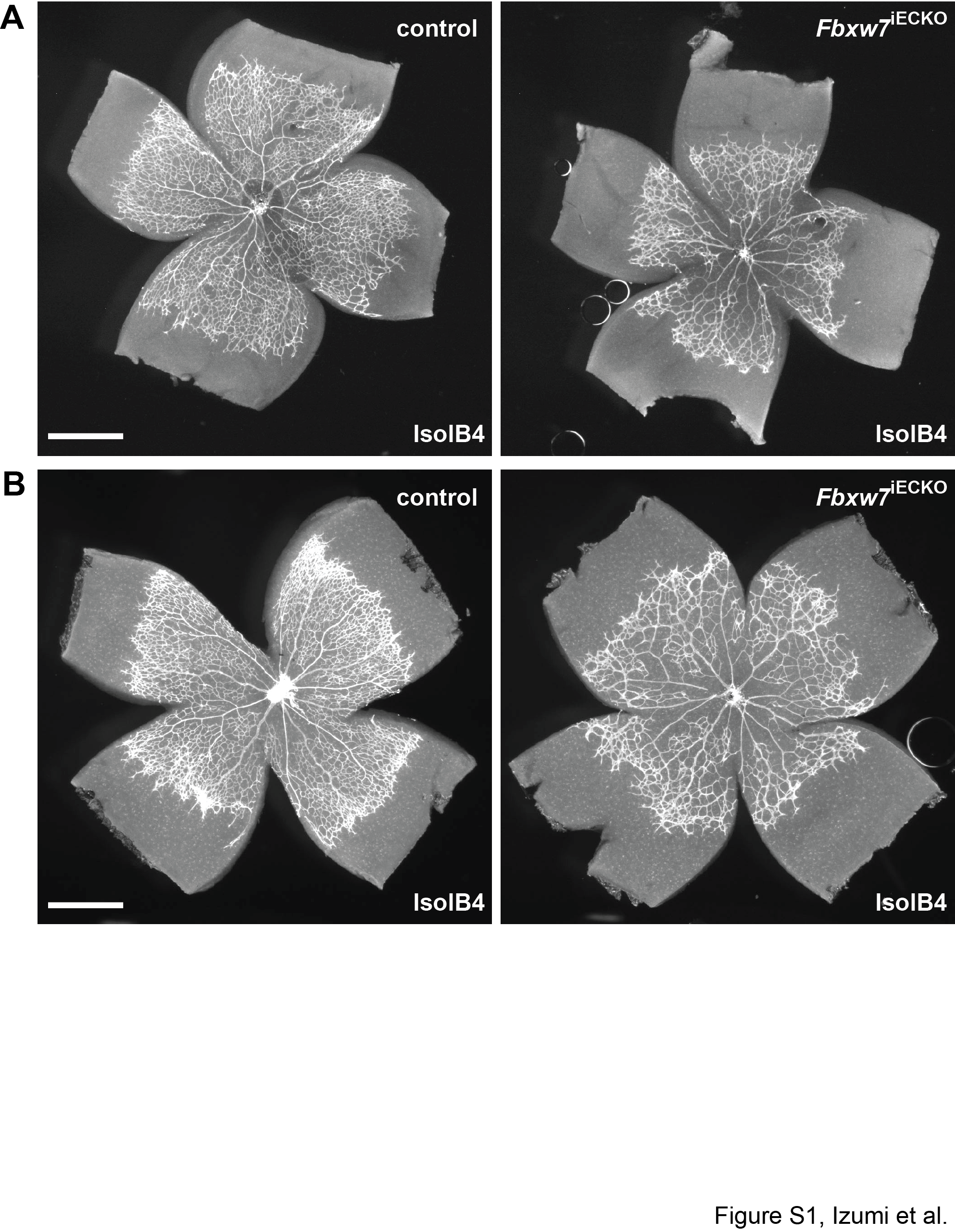

Supplement: Figure S1 — Defective retinal angiogenesis in Fbxw7 loss-of-function mutants. Confocal images of whole-mount Isolectin B4-stained retinas from P6 Fbxw7 iECKO mutants generated with the Cdh5(PAC)-CreERT2 (A) or Pdgfb-iCre (B) transgenic line, respectively. Controls (left panels) are respective littermates. Note the impaired retinal angiogenesis in the absence of endothelial Fbxw7. Scale bar is 500 µm. (TIF) [file pone.0041116.s001.tif]

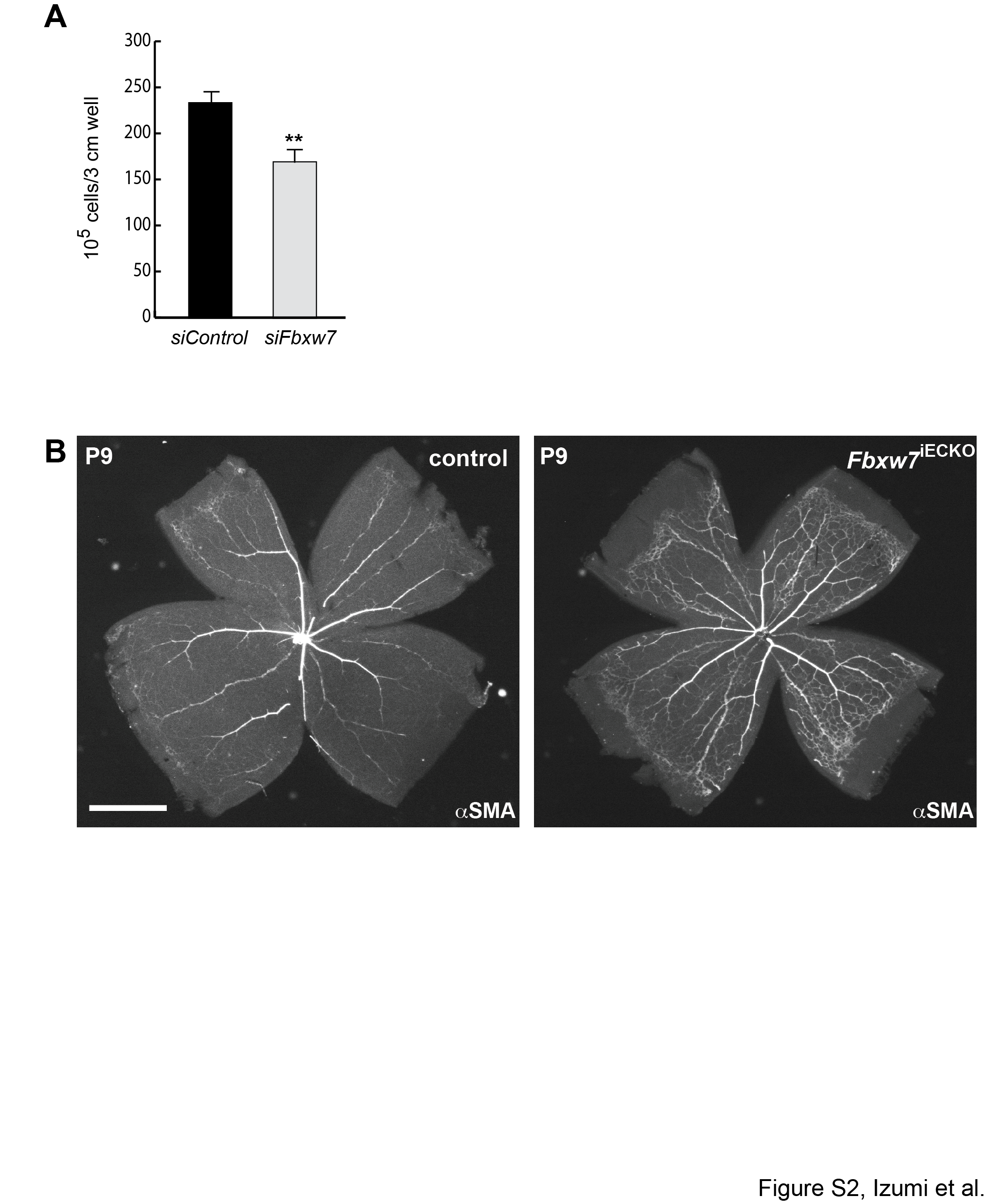

Supplement: Figure S2 — Fbxw7 controls HUVEC proliferation and smooth muscle cell recruitment. Quantitation of cell numbers in cultured siFbxw7 (Fbxw7 silenced) or siControl HUVECs, as indicated, at 72 hours after siRNA transfection (A). Error bars indicate SEM. P value (**) is <0.001. Confocal image of P9 whole-mount Fbxw7 iECKO (right) and control (left) retinas after staining with anti-smooth muscle actin (SMA) antibody (B). Note increased SMA signal in the mutant vasculature. Scale bar represents 500 µm. (TIF) [file pone.0041116.s002.tif]

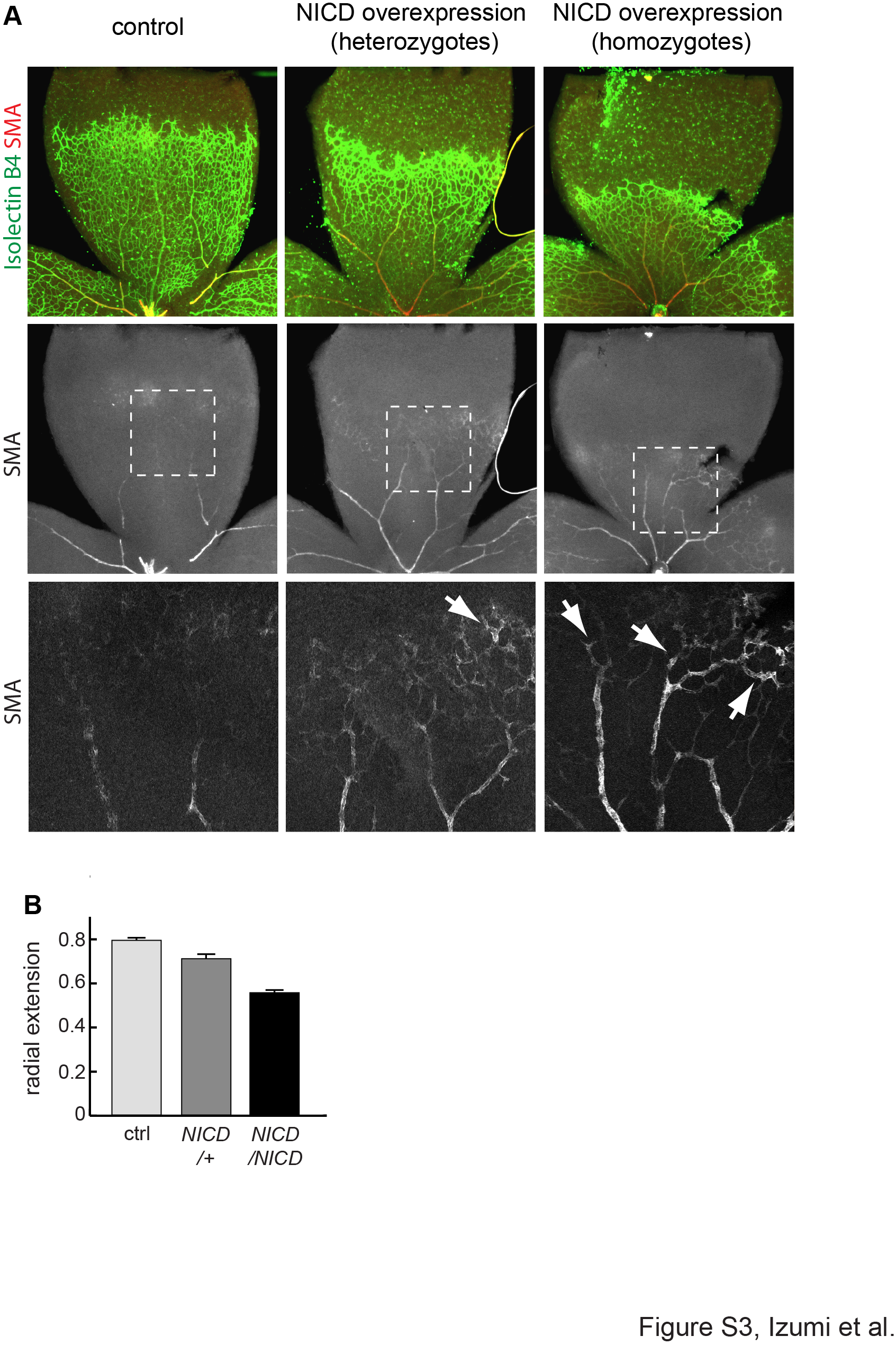

Supplement: Figure S3 — Endothelial overexpression of NICD phenocopies Fbwx7 defects. Confocal images of P6 control and endothelial cell-specific NICD gain-of-function retinas (A). Pdgfb-iCre transgenics were combined with Gt(ROSA)26Sor tm1(Notch1)Dam/J heterozygous (middle column) or homozygous mice (right column). ECs were visualized by Isolectin B4 (green), smooth muscle cells by αSMA immunofluorescence. Bottom panels show higher magnification of insets in the middle row. Arrows indicate extension of αSMA staining into peri-arterial capillary beds. Radial extension of the vascular plexus towards the periphery (B) was reduced in NICD/+ heterozygotes and NICD/NICD homozygotes. (TIF) [file pone.0041116.s003.tif]

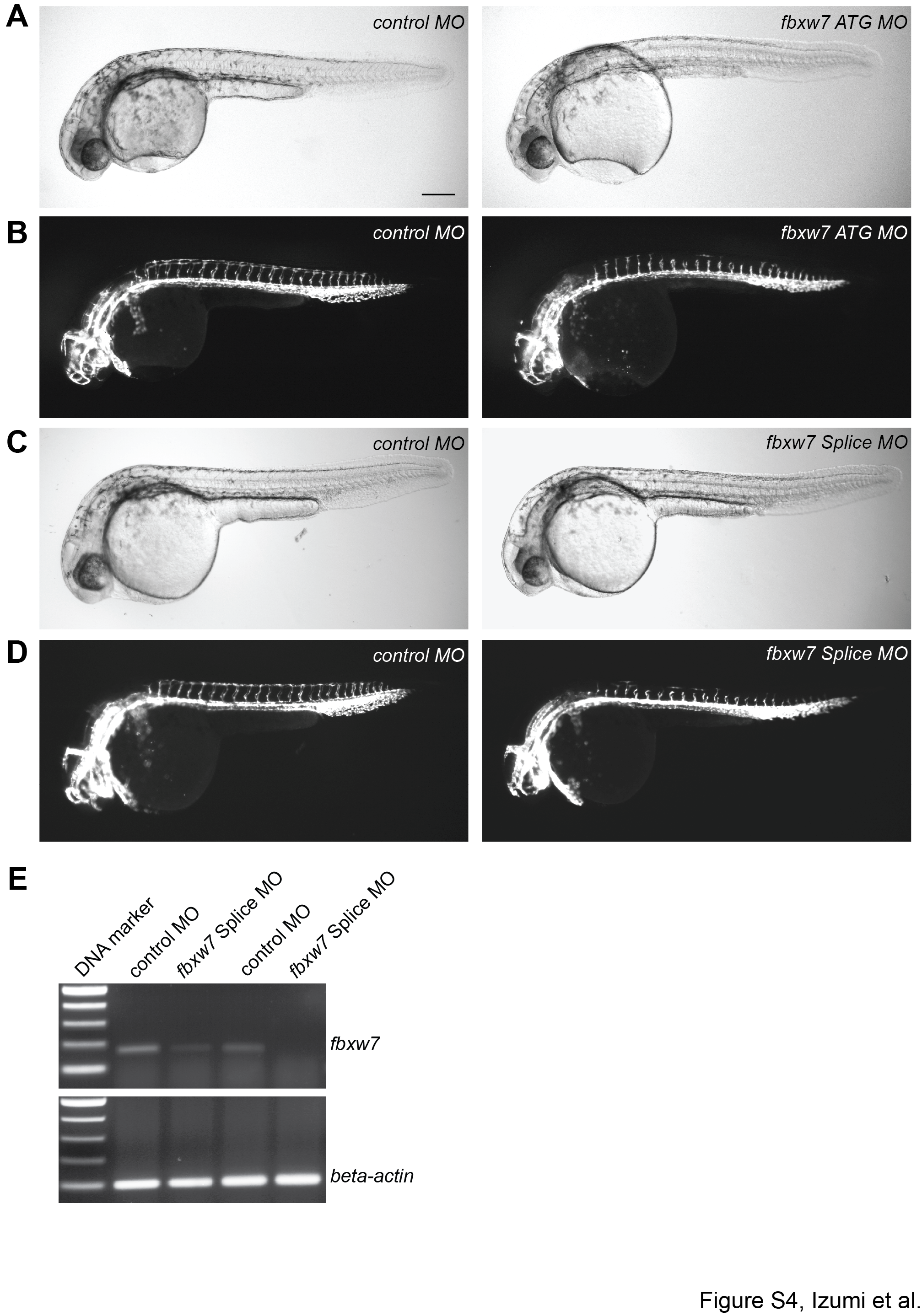

Supplement: Figure S4 — Vascular defects caused by the knockdown of zebrafish fbxw7 . Bright-field images (A, C) and endothelial fluorescence (B, D) of Tg(kdrl:EGFP)s843 zebrafish embryos at 32 hpf injected with control (control MO), fbxw7 translation-blocking (ATG MO) or fbxw7 splicing-blocking (Splice MO) morpholinos, as indicated. The knockdown of fbxw7 impaired ISV outgrowth and prevented the formation of the DLAV, while the size and general growth of the morphant embryos were unaffected. Scale bar is 200 µm. PCR analysis (E) showing the reduction of fbxw7 transcripts in zebrafish embryos injected with Splice MO in two independent experiments. Beta-actin PCR products were used as loading control. (TIF) [file pone.0041116.s004.tif]

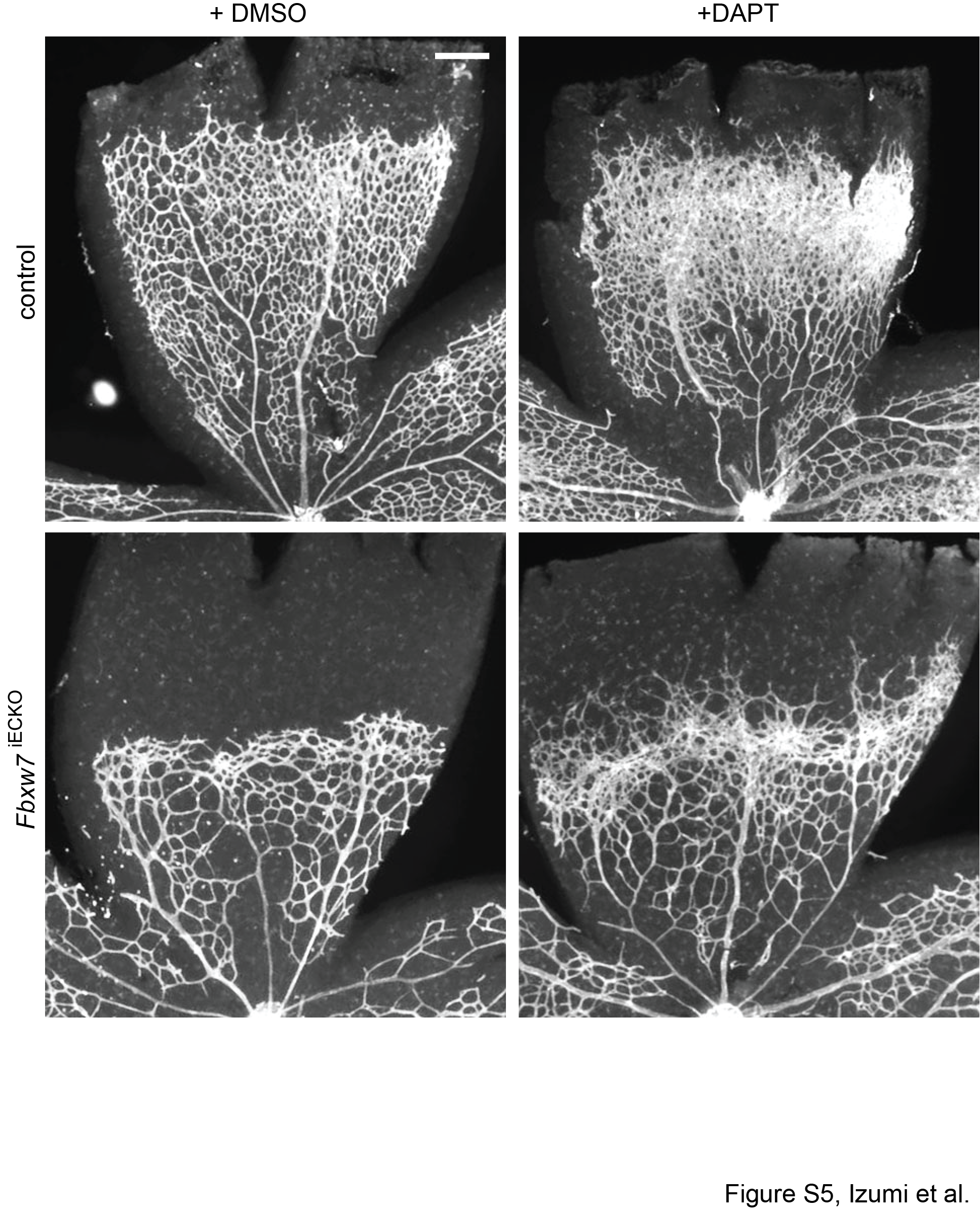

Supplement: Figure S5 — Notch inhibition restores vascular growth in Fbxw7 mutants. Confocal images of whole-mount Isolectin B4-stained retinas. Notch inhibition was achieved by administration of the γ-secretase inhibitor DAPT, which interferes with Notch cleavage and signaling, for 48 hrs prior to the isolation of the retinas at P6. DAPT partially restored enhanced sprouting and proliferation in the Fbxw7 iECKO vasculature. In contrast, angiogenesis was not increased in vehicle (DMSO)-injected Fbxw7 iECKO mutants. The phenotype of DMSO or DAPT-treated control retinas is shown in the upper row. Scale bar is 200 µm. (TIF) [file pone.0041116.s005.tif]
